# In Vivo Prediction of Breast Muscle Weight in Broiler Chickens Using X-ray Images Based on Deep Learning and Machine Learning

**DOI:** 10.3390/ani14040628

**Published:** 2024-02-16

**Authors:** Rui Zhu, Jiayao Li, Junyan Yang, Ruizhi Sun, Kun Yu

**Affiliations:** 1 College of Information and Electrical Engineering, China Agricultural University, Beijing 100083, China; ruiz@cau.edu.cn (R.Z.); lijiayao@cau.edu.cn (J.L.); junyan_yang163@163.com (J.Y.); 2 Scientific Research Base for Integrated Technologies of Precision Agriculture (Animal Husbandry), The Ministry of Agriculture, Beijing 100083, China; 3 College of Animal Science and Technology, China Agricultural University, Beijing 100193, China

**Keywords:** weight prediction, deep learning, machine learning, X-ray, precision farming

## Abstract

**Simple Summary:**

The breast muscle weight of broilers is a key indicator in poultry production. The accurate and nondestructive measurement of broiler breast muscle weight can improve breeding and the precision management level of broiler farms. Therefore, this study proposed an efficient method for predicting the breast muscle weight of broilers in vivo which can automatically predict broiler breast muscle weight. The experimental results demonstrate the method’s accuracy and superiority. The proposed method streamlines the data collection process, improves measurement efficiency, and provides crucial data support for broiler breeding and precision management.

**Abstract:**

Accurately estimating the breast muscle weight of broilers is important for poultry production. However, existing related methods are plagued by cumbersome processes and limited automation. To address these issues, this study proposed an efficient method for predicting the breast muscle weight of broilers. First, because existing deep learning models struggle to strike a balance between accuracy and memory consumption, this study designed a multistage attention enhancement fusion segmentation network (MAEFNet) to automatically acquire pectoral muscle mask images from X-ray images. MAEFNet employs the pruned MobileNetV3 as the encoder to efficiently capture features and adopts a novel decoder to enhance and fuse the effective features at various stages. Next, the selected shape features were automatically extracted from the mask images. Finally, these features, including live weight, were input to the SVR (Support Vector Regression) model to predict breast muscle weight. MAEFNet achieved the highest intersection over union (96.35%) with the lowest parameter count (1.51 M) compared to the other segmentation models. The SVR model performed best (R^2^ = 0.8810) compared to the other prediction models in the five-fold cross-validation. The research findings can be applied to broiler production and breeding, reducing measurement costs, and enhancing breeding efficiency.

## 1. Introduction

Chicken breast has gained increasing popularity among consumers in the market due to its attributes of high protein content, low-fat calories, and affordable price [1,2,3]. Therefore, breeding broiler chickens with a high yield of breast muscle holds significant economic value, which requires accurate measurement of the breast muscle weight [4,5,6]. The conventional approach to broiler breeding infers the carcass performance of an individual to be cultivated by using the slaughter value from individuals within the same family [7,8]. However, such methods may consume a large amount of time, compromise animal welfare, and incur high economic costs [9]. Furthermore, this approach may be influenced by individual differences in the group and cannot retain the individuals with high breeding value among the slaughtered population [10]. Therefore, it is imperative to employ a nondestructive, accurate, and efficient broiler phenotypic estimation method to select individuals with elevated breeding values.

With the development of medical imaging technology, it is easy to nondestructively visualize the intricate internal structures of organisms with enhanced precision and resolution [11,12,13]. In early studies, researchers manually determined the cross-sectional area of the pectoral muscle in ultrasound images and combined it with live weight to construct a regression equation for predicting the breast muscle weight of broilers [9,14,15]. However, this manual method for determining the area is time-consuming and highly depends on the operator. Two recent studies on the breast muscle weight of broilers employed external body traits and breast muscle thickness determined by ultrasound images to develop machine learning models for breast muscle weight [7,8]. Nevertheless, the process of ultrasonic measurement is tedious, including feather separation, gel application, and probe positioning, which is not suitable for the automation of data collection. In contrast, X-ray technology can complete the scan within seconds after the broiler is simply fixed, without the need for additional operations. It is often employed for assessing the physiological condition of animal organisms [16,17,18,19]. In addition, many phenotypic traits of most organisms can also be measured or analyzed from X-ray images [20,21,22]. However, the level of automation in the aforementioned studies remains relatively low, as they still require human intervention for image analysis.

Recently, advancements in deep learning technology have provided a novel solution for medical image recognition [23]. Numerous deep learning-based medical image diagnostic assistance technologies have been developed to help physicians diagnose diseases and predict drug interactions [24,25,26,27,28]. Simultaneously, deep learning-based segmentation networks are widely utilized in the study of biological phenotypic traits. Melo et al. [29] replaced the tedious task of manually delimiting cattle rib-eye areas in ultrasound images using UNet++. Jeong and Sung [30] utilized the improved Attention U-Net to acquire mask images of the heart and thoracic vertebra from dog chest X-ray images, allowing for the automatic measurement of their length and area to calculate the adjusted heart volume index. Tempelaere et al. [31] employed U-Net to accurately classify every pixel in the X-ray images of pears, thereby obtaining the type, size, and location of internal fruit lesions. Chen et al. [32] developed MCC-Net to precisely segment the internal structure of rice seedling stems in X-ray scans and generate high-resolution images for phenotypic analysis. However, the running of deep learning models requires substantial storage and computational resources. The memory consumption of the aforementioned models is relatively high, making them less suitable for deployment on edge devices with limited computational resources in real farm environments. Hence, a lightweight model that preserves high segmentation accuracy will be more advantageous for practical application scenarios.

To tackle these problems, this study proposed a nondestructive, accurate, and efficient method for predicting the in vivo breast muscle weight of broilers using X-ray images and live weight. The method primarily comprises image segmentation, feature extraction, and weight prediction components. Firstly, a novel lightweight segmentation model called multistage attention enhancement fusion segmentation network (MAEFNet), was proposed for the automatic acquisition of pectoral muscle mask images from X-ray images of broilers. The encoder of the proposed model is the pruned MobileNetV3 [33], which is a lightweight convolutional neural network designed for mobile and embedded devices. In the decoder, two efficient attention modules, the Attention Refinement Module (ARM) [34] and the Coordinate Attention Module (CAM) [35], were introduced to enhance the model’s understanding of channel importance and spatial information, thus facilitating the forward propagation of effective features and excluding irrelevant features within the images. In addition, the Feature Fusion Module (FFM) [34] was integrated into the model for the fusion of features from different stages, which will improve the model’s capability to recognize target regions of varying sizes. Next, 15 related shape features were extracted from the pectoral muscle mask images obtained with MAEFNet. Meanwhile, inspired by the previous studies on breast muscle weight [7,8], live weight was also included as one of the features for breast muscle weight prediction. To further enhance the model’s accuracy and reliability, the recursive feature elimination (RFE) method was employed to screen these features. Finally, these selected features were inputted into the Support Vector Regression (SVR) model to complete the prediction of breast muscle weight. The proposed method enables the cost-effective and efficient in vivo prediction of breast muscle weight in broilers, providing crucial informational references for broiler breeding and precision management.

The principal objectives of this study were as follows: (1) to design a segmentation model with high accuracy and low memory consumption to automatically acquire pectoral muscle mask images of broiler chickens; (2) to select the optimal feature subset and validate the superiority of the SVR model for predicting breast muscle weight; and (3) to validate the feasibility of combining deep learning and machine learning methods with X-ray imaging for predicting in vivo pectoral muscle weight.

## 2. Materials and Methods

### 2.1. Overall Process

**Figure 1:**
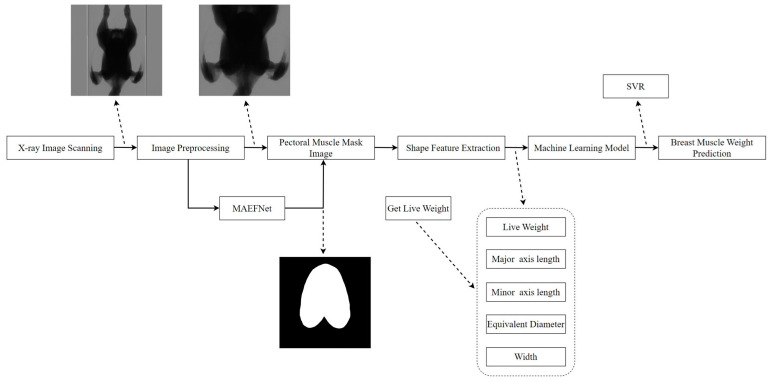
Overall process of the proposed method.

The overall process of the proposed method for breast muscle weight prediction is depicted in Figure 1. First, the X-ray images of the broilers were scanned, and then we weighed their live weight. Second, the X-ray images were preprocessed, including image cropping and scaling. Third, pectoral muscle mask images were obtained using MAEFNet. Fourth, relevant shape features were extracted from these mask images and integrated with live weight as inputs to the SVR model. Finally, the weight of the breast muscle was predicted.

### 2.2. Data Acquisition

The broilers used in this study were collected from a broiler farm in Guangze Country, Nanping City, Fujian Province. All broilers were reared in floor pens and freely administered water and food under the same recommended environmental and nutritional conditions (Feeding Standard of Chicken, China, NY/T 33-2004). The X-ray images were acquired with a customed mobile X-ray scanning device made by Aoying Testing Technology Co., Ltd. (Shanghai/Ningbo, China). 

**Figure 2:**
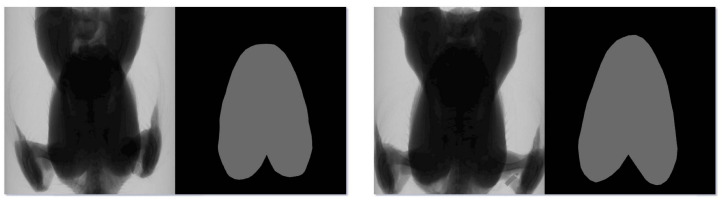
Annotated images of two broilers.

The broilers were positioned with their breasts directly facing the flat-panel detector (front-facing) during scanning. Concurrently, to increase the number of original images, data collection in the latter stages also included scans of broilers oriented with their backs facing the flat-panel detector. In addition, the scanning postures of broilers were checked before actually confirming the image. A total of 622 original X-ray images with a resolution of 1536 pixels × 1536 pixels were collected. These images were randomly divided into a training set, a validation set, and a test set in a ratio of 7:2:1. These images constituted the broiler X-ray breast muscle segmentation dataset and were used in the segmentation experiments. To annotate the broiler breast muscle region, the people involved in labeling learned the in vivo shape of the broiler breast muscle and the pixel distribution characteristics in the X-ray images. In addition, relevant professionals reviewed and modified the contents of the initial labeling to ensure the accuracy of the annotation. LabelMe software was employed to annotate the breast muscle regions in these 622 X-ray images. Figure 2 displays the annotation results of two broilers.

**Figure 3:**
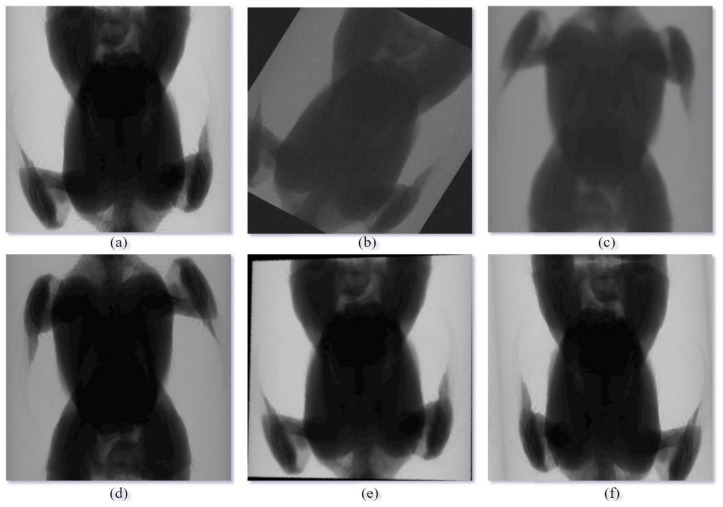
X-ray image augmented example. (**a**) Original; (**b**) Random rotation; (**c**) Vertical flip; (**d**) Horizontal flip after vertical flip; (**e**) Perspective transform; (**f**) Elastic transform after horizontal flip.

Data augmentation was performed using Albumentations [36] to enhance the robustness and generalization of the segmentation model. Figure 3 shows an example of augmented images.

In addition, a total of 100 front-facing acquisition broiler images were selected from these X-ray images. These images came from the validation and testing sets of the segmented dataset and corresponded one-to-one to actual broiler samples, meaning that 100 images represented 100 different broilers. After scanning these 100 broilers, their live weights were weighed, and they were subsequently dissected to obtain their actual breast muscle weights. These data were utilized in the breast muscle weight prediction experiments. All of the animal procedures were approved by the Experimental Animal Welfare and Animal Experiment Ethics Review Committee of China Agricultural University.

### 2.3. Data Preprocessing

Owing to the irrelevant background pixels within the original X-ray images and the high resolution of the primitive images, they would increase the recognition difficulty, memory consumption, and training time. Therefore, the X-ray images were cropped to remove irrelevant regions and scaled to a resolution of 512 pixels × 512 pixels.

### 2.4. MAEFNet Segmentation Model

**Figure 4:**
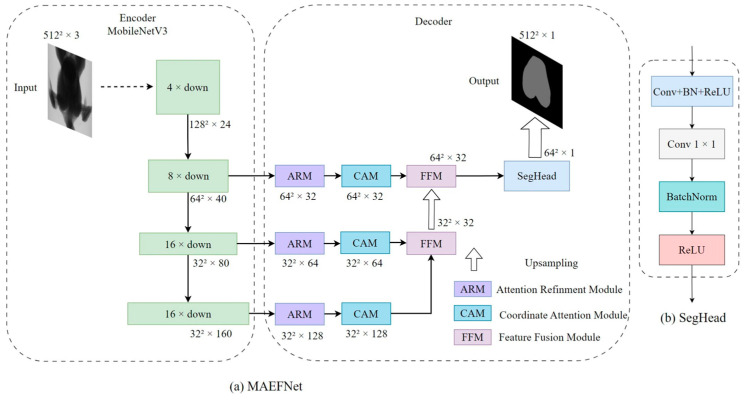
Architecture of MAEFNet. The thickness of the encoder block represents the spatial size, while the length indicates the number of channels. The thickness of the hollow arrow represents the magnification of upsampling.

To maintain excellent segmentation accuracy while reducing model memory consumption, a lightweight multistage attention enhancement fusion segmentation network (MAEFNet) was proposed for the automatic acquisition of pectoral muscle mask images. This network employs an encoder–decoder architecture and offers easy deployment and applicability in real-world environments. The pruned MobileNetV3 was employed as the encoder to efficiently capture features. Simultaneously, a novel decoder architecture was designed to achieve superior performance in semantic segmentation tasks. The detailed architecture of MAEFNet is depicted in Figure 4.

#### 2.4.1. Encoder

MobileNetV3 [33] is a lightweight deep neural network architecture designed for efficient feature extraction from images. It utilizes the inverted residual structure as the core component to capture and process features while reducing the computational cost. In MAEFNet, to alleviate the loss of local information caused by frequent downsampling, the stride of the last convolutional downsampling in MobilNetV3 was modified, which resulted in a reduction in the downsampling ratio from 32 to 16. In addition, the structure after this layer was discarded, further reducing the memory consumption of the model.

#### 2.4.2. Decoder

**Figure 5:**
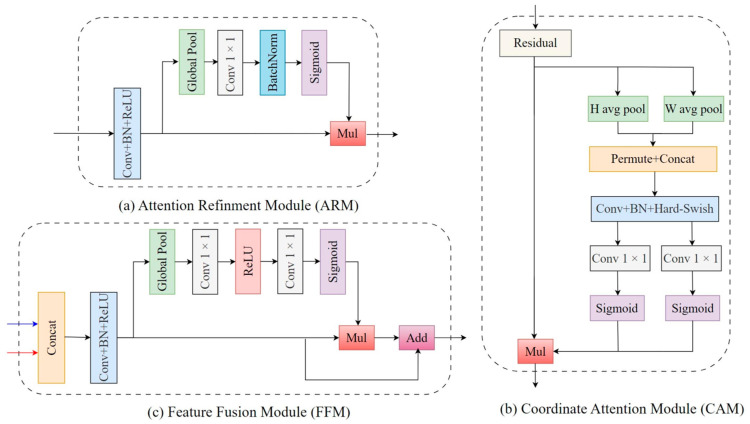
Architecture of (**a**) ARM; (**b**) CAM; and (**c**) FFM.

A novel decoder architecture was designed for the efficient enhancement and fusion of features at various stages of the encoder. The essential components of this decoder included the Attention Refinement Module (ARM) [34] for channel importance enhancement, the Coordinate Attention Module (CAM) [35] for spatial information enhancement, and the Feature Fusion Module (FFM) [34] for multistage features fusion. The design of these modules focused on achieving effective feature processing while maintaining a relatively low computational cost. The detailed structures of these three modules are shown in Figure 5.

Initially, feature maps at different stages in the encoder were compressed and refined by the ARM. This process adaptively recalibrated the importance of distinct channels within each feature map, thus enhancing the model’s sensitivity and discriminative capability towards critical information.

Specifically, first, a 3 × 3 convolutional transformation layer was applied to the input feature map $X_{input}$, yielding a channel dimension-reduced feature map denoted as $X^{\prime }\in {\mathbb{R}}^{C\prime \times H\times W}$. This convolutional transformation layer was composed of convolution, batch normalization, and an activation function. Then, a global average pooling layer aggregated each channel of $X^{\prime }$ into a vector to squeeze the global spatial information. Next, a 1 × 1 convolutional conversion layer was employed on the vector to derive the channel-wise attention weight denoted as $\alpha_{c}\in {\mathbb{R}}^{C\prime \times 1\times 1}$. This weight enabled the network to focus more on effective features without increasing excessive computational resources. Last, the final output $X_{output}\in {\mathbb{R}}^{C\prime \times H\times W}$ was obtained as the product of $X^{\prime }$ and $\alpha_{c}$. The detailed structure is depicted in Figure 5a and the computational process is shown in Equation (1).

(1) $$\begin{array}{l} X^{\prime }=\sigma (BN(f_{3\times 3}(X_{input})))\\ \alpha_{c}=\delta (BN(f_{1\times 1}(P_{Avg}(X^{\prime }))))\\ X_{output}=X^{\prime }\times \alpha_{c} \end{array}$$

where $f_{3\times 3}$ denotes a convolution operator with a kernel size of 3 × 3, $f_{1\times 1}$ denotes a convolution operator with a kernel size of 1 × 1, BN denotes the batch normalization operator, σ is the ReLU activation function, δ is the Sigmoid activation function, and $P_{avg}$ denotes a global average pooling operator.

Subsequently, the CAM, as an efficient attention mechanism, can enhance the spatial position information in feature maps with little computation, which is particularly suitable for embedding into a lightweight network to improve performance. It was introduced to compensate for the neglect of spatial details by the ARM.

Specifically, first, the input feature map, $X_{input}\in {\mathbb{R}}^{C\times H\times W}$, was globally averaged in the vertical and horizontal directions using two separate global average pooling layers, yielding two direction-aware feature maps. This step enabled the CAM to collect long-range dependencies in one spatial direction while preserving exact positioning information in the other direction, allowing the network to locate the regions of interest more precisely. Then, these two aggregated feature maps were concatenated in the spatial dimension and fed into a 1 × 1 convolutional conversion layer, generating an intermediate feature map $F\in {\mathbb{R}}^{C/r\times (H+W)\times 1}$. F encodes spatial information in two directions. Next, F was decomposed in the spatial dimension into two components, $F^{h}\in {\mathbb{R}}^{C/r\times H\times 1}$ and $F^{w}\in {\mathbb{R}}^{C/r\times 1\times W}$. Afterward, each of these components underwent individual processing through two 1 × 1 convolutional conversion layers, restoring the number of channels to match the input. This operation yielded the vertical and horizontal coordinate attention weights, $\alpha_{H}\in {\mathbb{R}}^{C\times H\times 1}$ and $\alpha_{W}\in {\mathbb{R}}^{C\times 1\times W}$, which can enable the model to focus more on the target areas. Last, these two attention weights were multiplied with the original input feature map, $X_{input}$, to produce the final output, $X_{output}\in {\mathbb{R}}^{C\times H\times W}$. The detailed structure is depicted in Figure 5b, and the computational process is shown in Equation (2).

(2) $$\begin{array}{l} F=\varsigma_{h}(BN(f_{1\times 1}(Concat_{s}(P_{Avg}^{H}(X_{input}),P_{Avg}^{W}(X_{input})))))\\ F^{h},F^{w}=Split(F)\\ \alpha_{H}=\sigma (f_{1\times 1}(F^{h})\\ \alpha_{W}=\sigma (f_{1\times 1}(F^{w}))\\ X_{output}=X_{input}\times \alpha_{H}\times \alpha_{W} \end{array}$$

where $\varsigma_{h}$ is the Hard Swish activation function, $P_{Avg}^{H}$ denotes the global average pooling operator with a kernel size of H × 1, $P_{Avg}^{W}$ denotes the global average pooling operator with a kernel size of 1 × W, $Concat_{s}$ denotes the concatenation operation along the spatial dimension, and Split denotes the decomposition operation along the spatial dimension.

Finally, fusing feature maps from different stages allows the model to acquire multiscale information, which is crucial for recognizing objects of varying scales. Meanwhile, it can partially compensate for the feature loss during the downsampling process, thus improving the model’s performance. Therefore, the FFM was introduced to efficiently integrate features from different hierarchical levels, resulting in a more comprehensive feature representation.

Specifically, the process began by concatenating the input feature maps, $X_{1}\in {\mathbb{R}}^{C_{1}\times H\times W}$ and $X_{2}\in {\mathbb{R}}^{C_{2}\times H\times W}$, along the channel dimension, yielding a fusion feature map, $X\in {\mathbb{R}}^{(C1+C2)\times H\times W}$, that combines information from two stages. Then, a channel dimension-reduced feature map, $X^{\prime }\in {\mathbb{R}}^{C\times H\times W}$, was generated through a 3 × 3 convolutional transformation layer. Next, a global average pooling operator and two 1 × 1 convolution operators, each followed by an activation function, were applied to produce the channel weight $\alpha_{c}\in {\mathbb{R}}^{C\times 1\times 1}$, which fully collected the channel-wise dependencies. Last, the final fused feature map, $X_{output}\in {\mathbb{R}}^{C\times H\times W}$, was obtained by adding the product of $X^{\prime }$ and $\alpha_{c}$ to the original $X^{\prime }$. The detailed structure is depicted in Figure 5c, and the computational process is shown in Equation (3).

(3) $$\begin{array}{l} X^{\prime }=\sigma (BN(f_{3\times 3}(Concat_{c}(X_{1},X_{2}))))\\ \alpha_{c}=\delta (f_{1\times 1}(\sigma (f_{1\times 1}(P_{Avg}(X^{\prime })))))\\ X_{output}=X^{\prime }+X^{\prime }\times \alpha_{c} \end{array}$$

where $Concat_{c}$ denotes the concatenation operation along the channel dimension.

#### 2.4.3. Setup

MAEFNet was trained from scratch. The parameters for network training are outlined in Table 1.

The cross-entropy loss function was employed on the training set to guide the model’s iterative updates. Meanwhile, the dice loss function was used to calculate the model’s loss on the validation set for a more intuitive interpretation of segmentation performance. The equations for these loss functions are provided as follows:

(4) $$CrossEntropyLoss=-\frac{1}{N}\sum_{i=1}^{N}\sum_{c=1}^{C}y_{i,c}log(p_{i,c})$$

(5) $$DiceLoss=1-\frac{2\left|A\cap B\right|}{\left|A\right|+\left|B\right|}$$

where N represents the total number of samples in the dataset; C represents the number of classes; $y_{i,c}$ is a binary indicator of whether class c is the correct classification for the i-th sample, with values of either 0 or 1; and $p_{i,c}$ is the predicted probability that the i-th sample belongs to class c, with values in the range [0-1]. A and B represent the number of pixels in the predicted and real target areas, respectively.

### 2.5. Breast Muscle Weight Prediction Model

The pectoral muscle mask images can be automatically obtained through MAEFNet, but they cannot be directly used to predict breast muscle weight. As a result, the extraction of shape features from the mask images and the construction of a machine learning model are needed to achieve the prediction of breast muscle weight. In addition, live weight is included as one of the features in the breast muscle weight prediction model. The reasons for this are listed as follows: (1) it is frequently employed in research on prediction methods for broiler breast muscle weight [7,8,15]; (2) it is highly correlated with breast muscle weight in poultry [37]; and (3) it is relatively easy to obtain.

Inspired by research that utilized computer vision technology to estimate weight [38], a multitude of shape features were extracted from pectoral muscle mask images. Table 2 provides detailed descriptions of the extracted shape features and explains their calculation principles, where *A* and *Ca* are the number of pixels in the pectoral muscle region and the convex hull of the pectoral muscle region, respectively.

The initial features of the breast muscle weight prediction model include live weight and 15 extracted pectoral muscle mask image shape features. Directly applying these features may lead to a decrease in the accuracy and reliability of the prediction model. Selecting appropriate features not only reduces data dimensionality to save computational resources but also suppresses data noise to alleviate model overfitting. Recursive feature elimination (RFE) is a common feature selection method, typically used to enhance the model’s accuracy and interpretability. The fundamental idea behind RFE is to iteratively build models and eliminate the least important feature at each step until reaching the predefined stop criterion. The ranking criteria of MRE is the coefficient of determination for the SVR model. RFE is firstly used to filter out the optimal subset containing different numbers of features. Then, the highest result among them is selected, including live weight, area, height, width, and diameter.

SVR (Support Vector Regression) is a variant of the SVM (Support Vector Machine) for regression tasks, falling under the category of supervised machine learning algorithms. The fundamental concept behind SVR is to map the input data into a high-dimensional feature space using a kernel function [39]. Subsequently, within this space, a hyperplane is constructed to be as close as possible to all data points while maintaining a certain tolerance for errors between predicted and observed values. The SVR model exhibits robustness against outliers and noise, and its inherent regularization parameters mitigate the risk of overfitting, making it suitable for data of varying dimensions [40]. Compared to other machine learning models, this model demonstrates exceptional generalization, and it excels in adapting to small-sample data [41]. Meanwhile, some related studies have achieved promising experimental results using this model [7,42,43]. Furthermore, the SVR model with linear kernel function outperformed both polynomial and radial kernel functions in preliminary experimental results. Therefore, SVR with a linear kernel was selected for predicting the breast muscle weight. The other parameters of the SVR model were determined through a grid search method with 5-fold cross-validation. Specifically, the penalty parameter was set to 10 and epsilon was set to 0.3, while the other parameters were maintained at their default values.

### 2.6. Experimental Settings and Evaluation Metrics

All experiments were conducted on the same experimental platform. The specific configuration of the experimental platform is displayed in Table 3. PyTorch was used to construct the segmentation models. OpenCV was employed to preprocess the raw images and extract features from mask images. Scikit-learn was adopted to build the machine learning models.

In the segmentation experiments, the precision (Pre), recall (Rec), intersection over union (IoU), and Dice similarity coefficient (DSC) were employed to evaluate the accuracy of the model. Pre measures the accuracy of the model in predicting target regions. Recall assesses the model’s ability to capture all parts of the actual target areas. The IoU is the ratio of the intersection to the union between the predicted results and the actual labels, reflecting the model’s capability for accurate identification of target areas. The DSC quantifies the degree of overlap between the model’s predictions and the actual labels. Additionally, the memory consumption and speed of the model were assessed according to the number of parameters (Params) and the inference time for a single image (Latency). The equations for Pre, Rec, IoU, and DSC are defined as follows:

(6) $$Pre=\frac{TP}{TP+FP}\times 100\%$$

(7) $$Rec=\frac{TP}{TP+FN}\times 100\%$$

(8) $$IoU=\frac{TP}{TP+FP+FN}\times 100\%$$

(9) $$DSC=\frac{2TP}{2TP+FP+FN}\times 100\%$$

where TP is the number of pixels in the target area that were correctly predicted, FN is the number of pixels in the target area that were incorrectly predicted as the nontarget area, TN is the number of pixels in the nontarget area that were correctly predicted, and FP is the number of pixels in the nontarget area that were incorrectly predicted as the target area.

In the weight prediction experiments, the coefficient of determination (R^2^), root mean square error (RMSE), mean absolute error (MAE), and mean relative error (MRE) were used to evaluate the prediction models. R^2^ measures the degree of fit between the model’s predictions and the actual values. RMSE calculates the average magnitude of error between predicted and actual values. MAE calculates the average absolute difference between predicted and actual values. MRE measures the average relative difference between the predicted and actual values, expressed as a percentage. Their equations are defined as follows.

(10) $$R^{2}=1-\frac{\sum_{i=1}^{N}{\left(\hat{y}-y_{i}\right)}^{2}}{\sum_{i=1}^{N}{\left(y_{i}-\bar{y}\right)}^{2}}$$

(11) $$RMSE=\sqrt{\frac{1}{N}\sum_{i=1}^{N}{\left({\hat{y}}_{i}-y_{i}\right)}^{2}}$$

(12) $$MAE=\frac{1}{N}\sum_{i=1}^{N}\left|{\hat{y}}_{i}-y_{i}\right|$$

(13) $$MRE=\frac{1}{N}\sum_{i=1}^{N}\left|\frac{{\hat{y}}_{i}-y_{i}}{y_{i}}\right|\times 100\%$$

where N denotes the total number of samples, $y_{i}$ denotes the actual weight for the i-th sample, ${\hat{y}}_{i}$ denotes the predicted weight for the i-th sample, and $\bar{y}$ denotes the average actual breast muscle weight of the broilers.

## 3. Results

### 3.1. Comparison of Segmentation Models

To better validate the overall performance of the proposed MAEFNet, this study compared the experimental results of this model with those of five other models, including UNet [44], TransUNet [45], DeepLabV3Plus [46], BiSeNet [34], and LR-ASPP [33]. The training parameters and loss function of the compared models were kept consistent with MAEFNet.

The results of the segmentation experiments are shown in Table 4. In terms of segmentation accuracy, MAEFNet achieved a Pre, IoU, Rec, and DSC of 97.89%, 96.35%, 98.40%, and 98.15%, respectively, all of which were higher than those of the other compared models. Compared to LR-ASPP, which used the same encoder, MAEFNet had an improvement of 1.01% in the IoU, indicating that the designed decoder can better enhance the model’s performance. The IoU increased by 0.65% compared to UNet, which had the second-highest accuracy. In terms of memory consumption, MAEFNet had the lowest number of parameters (1.51 M), decreasing by 53% and 87% compared to LR-ASPP and BiSeNet, respectively, and this was significantly lower than the other compared models. In terms of inference speed, a single image inference time of MAEFNet was approximately 4.75 milliseconds (ms), slightly longer than that of LR-ASPP (3.68 ms) and BiSeNet (4.01 ms) but significantly lower than other compared models.

**Figure 6:**
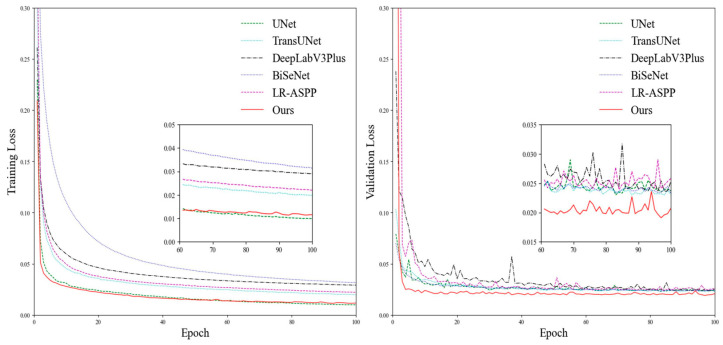
Training and validation loss curves of different segmentation models.

The loss curves for different models on the training and validation sets are depicted in Figure 6. All models exhibited a gradual reduction in loss on the training set as the epoch increased, eventually reaching a stable level. For the validation set, MAEFNet converged faster compared to the other models. Furthermore, after the 10th epoch, MAEFNet exhibited lower loss values, which indicates that MAEFNet performs better on new images and possesses good generalization capabilities.

**Figure 7:**
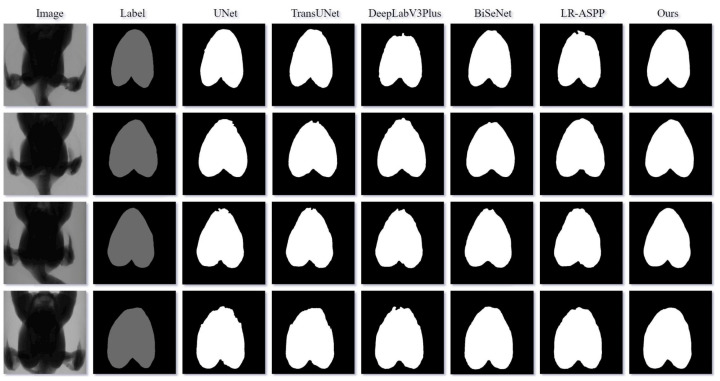
Visualized segmentation results of different segmentation models.

Figure 7 displays the visualized segmentation results of different models. LR-ASPP and DeepLabV3Plus exhibited rough predictions of the top boundary of the pectoral muscle, with some mis-segmentation issues in the bottom boundary. UNet, TransUNet, and BiSeNet performed slightly better but still had varying degrees of mis-segmentation at the top boundary. In contrast, MAEFNet can precisely identify the top and bottom boundaries of the pectoral muscle and predict a clear and complete contour. This demonstrates the model’s ability to effectively extract edge features from images, resulting in excellent segmentation results.

In summary, MAEFNet achieved precise segmentation of the breast muscle area in broiler X-ray images, with low memory consumption while maintaining an acceptable speed. These attributes significantly enhance the model’s adaptability and usability in real farm environments with constrained computational resources, providing robust support for its swift deployment and practical application.

### 3.2. Comparison of Weight Prediction Models

To adequately validate the superiority of the SVR model in predicting breast muscle weight, this study compared it with other prevalent machine learning models, including K-Nearest Neighbor (KNN), Decision Tree (DT), Random Forest (RF), Adaptive Boosting (AdaBoost), and Extreme Gradient Boosting (XGBoost). The hyperparameters of all of these machine learning models were determined through the grid search approach with 5-fold cross-validation.

The experimental results of the prediction of breast muscle weight in this study were the average of 5-fold cross-validation. Table 5 presents the results of different models before and after feature selection. It can be observed that the SVR model performed better than other models, regardless of whether feature selection was used. Notably, after the feature selection, all models exhibited varying degrees of optimization across all metrics, especially with SVR’s R^2^ showing an improvement of 0.0381. This amply illustrates the role of feature selection in enhancing model accuracy. Subsequent analysis is based on the feature selection.

The SVR model demonstrated both a satisfactory actual fitting degree and prediction error in predicting breast muscle weight, with an R^2^ of 0.8810, an RMSE of 25.38 g, an MAE of 20.48 g, and an MRE of 3.668%. Compared with KNN, DT, RF, ADBoost, and XGBoost, the SVR model achieved a higher R^2^ by 0.0919, 0.1271, 0.0401, 0.0452, and 0.0826, respectively. Similarly, in terms of the MAE indicator, the SVR model displayed reduced values of 6.25, 10.20, 3.72, 3.97, and 6.56 compared to these models, respectively.

**Figure 8:**
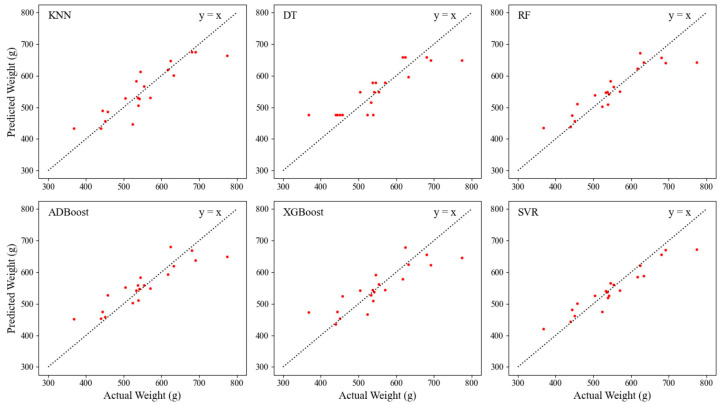
Scatter plots of predicted and actual values from different weight prediction models.

Figure 8 shows the distribution of actual values and predicted values of breast muscle weight for different prediction models based on the same test set. The error between the actual value and the predicted value is the difference between the abscissa and ordinate of the sample point. The degree of clustering of the sample points near the 45-degree diagonal line (y = x) reflects the fitting performance of the model. It can be seen that most of the sample points in the SVR model are closer to this diagonal line, which illustrates the advantage of the SVR model in predicting breast muscle weight.

In general, in light of the results obtained, the SVR model demonstrated a commendable fitting effect and prediction error in translating the selected features into the actual breast muscle weight of broilers. This indicates that the combination of MAEFNet and SVR models can effectively predict in vivo breast muscle weight. The accuracy meets the requirements and expectations of practical farm applications.

## 4. Discussion

### 4.1. Discussion of Data Processing

In this study, cropping and scaling were employed to process the original images. Cropping allows the model to focus more on the breast muscle area in the image while scaling alleviates memory consumption and computational costs during training and prediction. These steps contribute to improving the accuracy and efficiency of the model. However, they may also lead to some loss of details in the images. Even so, given the constrained computational resources in actual farm environments and the ultimate prediction results, these losses are deemed acceptable.

### 4.2. Discussion of the Evaluation Metrics

In the context of segmenting breast muscles in X-ray images for shape feature extraction, all four metrics—Pre, Rec, IoU, and DSC—are important and complementary. Pre and Rec provide insights into the model’s accuracy at identifying breast muscle pixels and the completeness of muscle segmentation, respectively. The IoU and DSC offer a more comprehensive evaluation by measuring the overall agreement between the predicted segmentation and the ground truth, which directly influences the reliability of the subsequent shape feature extraction.

For the weight prediction, R^2^ is useful for assessing how well the model captures the variability in the breast muscle weight. However, it cannot directly reflect the actual error of the prediction model. The MAE offers a simple and intuitive measure of the average error, while the RMSE provides insight into the average error magnitude, with a higher penalty for large errors. The MRE provides a relative measure of a model’s prediction accuracy, ensuring that evaluation results are not influenced by the scale of measurement.

For a robust evaluation, it is best to consider all of these metrics together, because they offer a comprehensive evaluation of a model’s accuracy and reliability in different tasks, with each metric contributing unique and valuable insight into the model’s performance.

### 4.3. Discussion of MAEFNet

By comparing the results in Table 4, the proposed MAEFNet outperformed other models in both segmentation accuracy and parameter count. In addition, it can be observed in Figure 7 that MAEFNet exhibited a comprehensive recognition capability for pectoral muscles of different sizes, especially in the top or bottom boundary, which is challenging for other segmentation models. On the one hand, the accuracy of the segmentation model influences the reliability of feature extraction, subsequently impacting the precision of the subsequent prediction model. On the other hand, the quantity of parameters in the model directly influences its memory consumption. Models with lower memory consumption can be more easily deployed in edge devices with limited computational resources, thereby reducing the usage costs of the model. MAEFNet possesses the advantages of low memory consumption and high accuracy, making it more suitable for application in practical farms with constrained computational resources.

Additionally, it is worth noting that UNet has fewer parameters compared to DeepLabV3Plus and TransUNet, yet its actual inference time was longer. Similarly, the proposed MAEFNet, despite having fewer parameters, was slightly slower in inference time compared to BiSeNet and LR-ASPP. The model’s inference speed is influenced by various factors, such as memory access, parallelism, and the software and hardware environments [47]. MAEFNet discards the final layers of the MobileNetV3 encoder, a pivotal factor contributing to its model parameter count being less than that of the LR-ASPP. Simultaneously, the decoder incorporates many pooling, batch normalization, and activation operations that do not increase the model’s parameter count but consume computational time. In contrast, LR-ASPP has an optimized quantity of channels and filters to enhance the computational efficiency [33]. This targeted optimization is a crucial factor enabling LR-ASPP to maintain a lower inference time despite having a higher parameter count.

Table 6 shows the performance after integrating different backbone networks into the model. It is observed that, whether for ResNet50, VGG16, or Xception, the pruned MobileNetV3 outperforms these three backbone networks in various metrics. The IoU metric increases by 1.58%, 2.68%, and 1.71%, respectively, while the number of parameters decreases by 94.33%, 82.15%, and 93.62%. This improvement can be attributed to a series of architectural optimization methods employed in MobileNetV3 and the introduction of depthwise separable convolution and the SE attention mechanism in the inverted residual structure. Furthermore, the decoder in MAEFNet is specifically optimized for the pruned MobileNetV3, while such optimization may not be suitable for other backbone networks.

The rationale behind choosing these improvement strategies is to enable the model to achieve a better balance between memory consumption and accuracy. In addition, to better assess the contributions of various improvement strategies to the model, ablation experiments were conducted by incrementally incorporating strategies into the original network, which is founded on the raw MobileNetV3 encoder and multistage feature extraction. Table 7 presents the results of different combinations of strategies, where PMobileNetV3 denotes MobileNetV3 with a modified downsampling rate. Before adjusting the downsampling rate in the encoder, the model’s IoU and DSC were 95.68% and 97.80%, respectively. Following the modification of the downsampling rate, these metrics improved by 0.15% and 0.09%, respectively. Upon the integration of the ARM and CAM, reinforcing crucial information in the model’s feature maps, the IoU and DSC improved by 0.34% and 0.18%, respectively. Lastly, with the inclusion of the FFM optimizing the feature fusion process, the model exhibited a notable enhancement of 0.67% and 0.35% in the two metrics compared to the initial values, reaching 96.36% and 98.15%.

**Figure 9:**
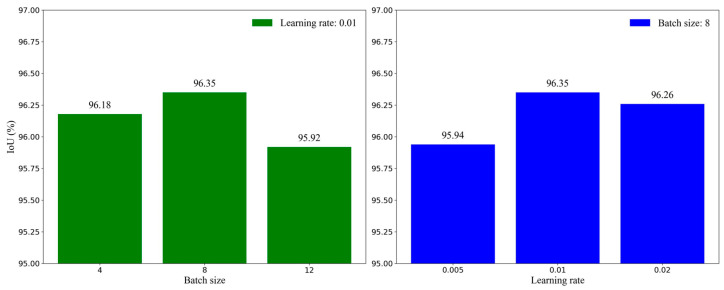
Comparison of the results with different hyperparameters.

Figure 9 shows the IoU scores of MAEFNet after training with different batch sizes and learning rates. It can be observed that the optimal results were achieved when the batch size was set to 8, and the learning rate is set to 0.01. In other cases, fixing one parameter while varying the other, whether increasing or decreasing the value of the parameter, caused a decrease in the IoU.

To better validate the generalizability of MAEFNet, a publicly available dataset of human lung X-ray images was introduced [48,49]. Table 8 presents the results of the different models based on this dataset. It can be observed that, although MAEFNet exhibited relatively poor performance regarding the Rec metric, it achieved comprehensive superiority in the Pre, IoU, and DSC metrics. Specifically, the IoU showed an improvement of 0.2%, 0.61%, 1.54%, 1.58%, and 2.58% compared to UNet, TransUNet, DeepLabV3Plus, BiSeNet, and LR-ASPP, respectively. A high Pre and low Rec suggest that the model has high confidence in lung region determination but tends to be conservative, resulting in a decrease in false positives and an increase in false negatives. A higher IoU and DSC indicate better consistency between the segmentation results predicted by MAEFNet and the actual results.

To further improve the performance and reduce computational costs, model compression methods can be considered. These methods include pruning, quantization, and knowledge distillation. Simultaneously, the inference speed of the model can be optimized by judiciously reducing the number of filters and channels in certain structures of MAEFNet. Additionally, semi-supervised learning can leverage both labeled and unlabeled data to enhance model training efficiency and performance, possibly through iterative training using self-training or pseudo-labeling methods. Unsupervised learning, on the other hand, can uncover hidden patterns or features within the data that may improve the understanding of the underlying processes and potentially boost model performance. Possible avenues include the use of autoencoders for feature extraction or generative adversarial networks (GANs) for data augmentation. These techniques achieve commendable segmentation performance with minimal manual annotations. The segmentation dataset labels in this study are manually annotated, and these techniques can be integrated to further enhance the overall efficiency of the proposed method.

### 4.4. Discussion of Selected Features

In this study, the features selected by RFE include live weight, area, height, width, and diameter. Live weight represents the overall body weight of broilers in a live state. Area directly reflects the size of the breast muscle. They are highly correlated with breast muscle weight and have been used in previous research [9,15]. The length and width of the breast muscle in vivo are similar to the extracted height and width from X-ray images, and their correlation with breast muscle weight has been validated in a previous study [5]. As for diameter, it represents the maximum distance from the center point of the breast muscle to the farthest edge, reflecting the shape of the breast muscle from another perspective. The combination of these features better captures the overall size and shape characteristics of the breast muscle.

### 4.5. Discussion of Weight Prediction Models

As shown in Table 5, the SVR model achieved a better fitting effect and lower prediction error compared to other models. This may be ascribed to the use of an epsilon-insensitive loss function, allowing the model to ignore errors within a small range and focus more on structural errors rather than random noise [50]. The poorer performance of the XGBoost model based on the test set may be attributed to its better suitability for large-scale datasets, which can entail a risk of overfitting or becoming trapped in local optima when applied to small datasets [51].

**Figure 10:**
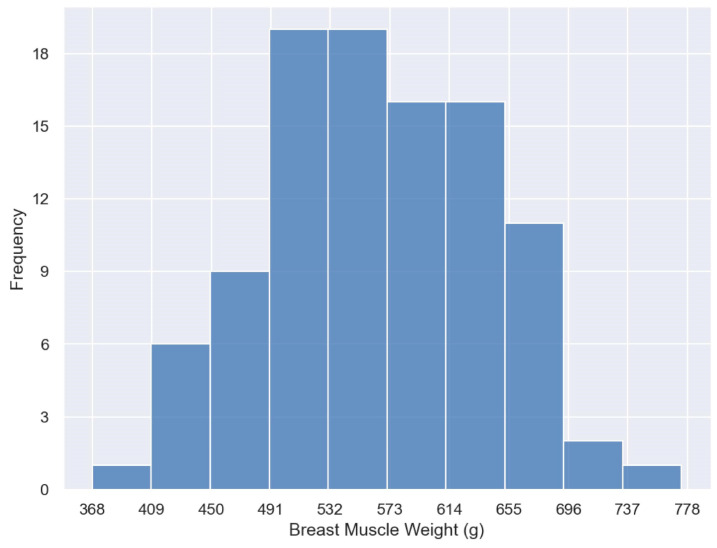
Distribution of broiler chicken breast muscle weights.

From Figure 8, it can be observed that the SVR model is more accurate in predicting the weight of samples within the range of 400–750 g. This may be attributed to the higher consistency in the developmental characteristics of the breast muscle within this weight range. Notably, all models exhibit similar prediction biases for samples with weights below 400 g and above 750 g. This may be caused by the imbalanced breast muscle weight distribution within the dataset [52]. Figure 10 illustrates the distribution range of breast muscle weights for the 100 broilers used in the weight prediction experiments. Samples weighing below 400 g and above 750 g were notably scarce, while the number of samples in the middle range was considerably higher. Therefore, the training set naturally contained more data belonging to the middle range, leading the models to become biased toward the middle range values when predicting these lighter or heavier breast muscle weight samples [53]. Furthermore, the developmental characteristics of breast muscle appearance may differ for samples with excessively high or low weights, and the limited quantity of samples hinders the model from adequately learning relevant features. Therefore, appropriately increasing the number of samples below 400 g and above 750 g would improve the performance of the prediction model.

### 4.6. Discussion of the Proposed Method

The proposed method may be the first attempt to predict the breast muscle weight of live broilers using conventional two-dimensional X-ray images. The X-rays generated by the machine with a kinetic energy of up to 5 MeV do not induce radioactivity in the exposed food [54]. Therefore, the use of X-ray detection for broiler chickens does not pose food safety concerns. Compared with the traditional measurement method, this method eliminates the need for slaughtering broilers, saving a lot of time and the cost of broilers and manpower required for slaughtering. In addition, the memory consumption of the proposed MAEFNet is very low, which further reduces the deployment cost of the model.

Previous studies have typically used computed tomography (CT), dual-energy X-ray absorptiometry (DEXA), and ultrasound techniques to assess the body composition of live broilers [7,8,9,15,55]. CT and DEXA provide more features for the target region, which may lead to more accurate prediction results. However, the machines are costly, the scanning process is time-consuming, and anesthesia is typically necessary before the broilers are scanned, which is not suitable for practical production. The advantages of ultrasound technology lie in its relatively low cost and absence of radiation exposure. Yet, the measurement process is cumbersome, including feather removal, gel application, and precise probe positioning. In addition, outcomes are susceptible to the influence of different operators [15]. Moreover, such methods frequently necessitate some additional body measurements [7,8], which can also be a time-consuming and laborious process.

In contrast, the X-ray technology used in this study can complete the acquisition of raw image data through simple fixation. It does not require anesthesia or other preparatory procedures, and the operation’s process is also simpler and faster. Compared to other medical imaging approaches, X-ray technology is more convenient, which greatly simplifies the acquisition process of raw data and improves the overall efficiency of measurement. Certainly, this technology has limitations, such as radiation exposure or image artifacts. Additionally, conventional X-ray technology may not be able to clearly observe certain visceral tissues within the animal’s body.

The time consumed for each step of our method is shown in Table 9. The first and second steps both contain the time for fixing broilers. The actual X-ray scanning duration for a single broiler is approximately 2 seconds (s). The fifth step contains the time for feature extraction. It can be seen that the breast muscle weight prediction of a single broiler can be completed in around 30 s. This is credited to the convenience of X-ray scanning and the autonomous learning capability of deep learning methods on the features of raw image data. Moreover, the majority of the time is taken up by the first two steps. In the future, the integration of a weight measurement device into the image acquisition equipment may be considered to shorten the overall time.

The samples in this study were randomly selected from broilers of different genders in the same broiler farm. Their weight distribution was close to a normal distribution. This is a common occurrence under natural conditions, indicating no deliberate selection bias. However, it should be acknowledged that the samples are all from the same farm, which may introduce potential biases.

The limitation of this study lies in solely verifying the effectiveness of the proposed method in predicting the weight of breast muscle in broilers. This may result in the selected five features being more suitable for predicting breast muscle weight. To better extend the proposed method to other slaughter traits in broilers, such as wings and legs, a similar roadmap to this study will be adopted. Firstly, MAEFNet will be utilized to obtain masks corresponding to different traits. Then, RFE will be employed to determine the optimal feature subsets for various slaughter traits. Finally, the SVR model will be used for weight prediction. In addition, future work also includes further training and validating the models involved in the method in a wider variety and quantity of poultry, which will further improve the generalizability of the proposed method.

Despite these shortfalls, the convenience and efficiency of the proposed method render it more practical for the large-scale nondestructive measurement of breast muscle weight in broilers, which is of great value for the breeding and precision management of broilers. In general, the proposed method strikes a favorable balance between cost, efficiency, and accuracy, catering to the needs of practical farm environments.

## 5. Conclusions

This study proposed a simple and effective method for the automated prediction of in vivo breast muscle weight in broilers from X-ray images. It integrates image segmentation, feature extraction, and weight prediction as its core components. Initially, a novel segmentation network, MAEFNet, was designed. This network achieved a satisfactory balance between accuracy and memory consumption (IoU = 96.35%, Params = 1.51 M) while preserving an acceptable inference speed (Latency = 4.75 ms). Subsequently, RFE was applied to refine features, including live weight and the extracted features from the mask image, effectively reducing prediction errors of breast muscle weight. Finally, the SVR model was adopted to transform the selected features into breast muscle weight. This method, ultimately, achieved an R^2^ of 0.8810, an RMSE of 25.38 g, an MAE of 20.48 g, and an MRE of 3.668% under five-fold cross-validation. The findings suggest that the integration of deep learning and machine learning techniques offers a viable approach for predicting the in vivo breast muscle weight of broilers through X-ray imagery. It can be applied to the production and breeding work in practical broiler farms.

The application of this method can assist farm caretakers In monitoring the nutritional status of breast muscle in a large-scale broiler population in less time, enabling the formulation of more precision management strategies over time. Furthermore, by accurately and swiftly measuring breast muscle weight, farm breeders can more easily cultivate individuals with superior breast muscle characteristics at reduced costs, thereby accelerating genetic gains and promoting the sustainable development of the poultry industry.

## Figures and Tables

**Table 1:** The training parameters of MAEFNet.

Parameters	Value
Epochs	100
Batch size	8
Learning rate	0.01
Optimizer	SGD
Momentum	0.9
Weight decay	0.0001

**Table 2:** The list of shape features extracted from the pectoral muscle mask image.

Feature	Description	Symbol
Area	Square root of the number of pixels in the pectoral muscle	$Ar=\sqrt{A}$
Convex area	Square root of the number of pixels in the convex hull of the pectoral muscle	$Co=\sqrt{Ca}$
Perimeter	Number of pixels in the pectoral muscle boundary	*P*
Major axis length	Major axis length of an ellipse enclosing the pectoral muscle	*Mal*
Minor axis length	Minor axis length of an ellipse enclosing the pectoral muscle	*Mil*
Height	Height of the pectoral muscle	*H*
Width	Width of the pectoral muscle	*W*
Rectangle area	Square root of the product of the pectoral muscle height and width	*Ra =* $\sqrt{H*W}$
Ellipticity	Ratio of the major axis length to the minor axis length	*El = Mal/Mil*
Diameter	Diameter of a circumscribed circle of the pectoral muscle	*D*
Equivalent diameter	Diameter of a circle equal to the area of the pectoral muscle	*Ed* = $2*\sqrt{A/\pi }$
Heywood circularity factor	Degree to which the pectoral muscle is close to the circle	*Hcf* = *P*/(2$*\sqrt{\pi *A}$ *)*
Aspect	Ratio of the height to the width of the pectoral muscle	*As = H/W*
Curvature	Ratio of the pectoral muscle area to the circumscribed circle area	*Cu* = 4∗*A*/(*π*∗*D*^2^)
Complexity	Ratio of a square of the perimeter to the pectoral muscle area	*Cl* = *P*^2^/*A*

**Table 3:** The configuration of the experimental platform.

Configuration	Parameter
Operating system	Windows 10
CPU	Intel Core i9-13900K
GPU	NVIDIA GeForce RTX 4090
Development language	Python 3.10
Framework	PyTorch 1.12 + OpenCV 4.8+ Scikit-learn 1.2
CUDA version	CUDA 12.1

**Table 4:** Performance of different segmentation models.

Model	Pre (%)	IoU (%)	Rec (%)	DSC (%)	Params (M)	Latency (ms)
UNet	97.66	95.70	97.95	97.84	17.26	13.26
TransUNet	97.42	95.52	98.00	97.73	77.37	11.19
DeepLabV3Plus	97.09	95.44	98.26	97.69	39.76	9.68
BiSeNet	97.57	95.56	97.89	97.76	11.89	4.01
LR-ASPP	97.38	95.34	97.85	97.65	3.22	3.68
Ours	97.89	96.35	98.40	98.15	1.51	4.75

**Table 5:** Comparison of different prediction models.

Model	Without Feature Selection				With Feature Selection			
	R^2^	RMSE	MAE	MRE (%)	R^2^	RMSE	MAE	MRE (%)
KNN	0.7833	35.11	27.02	4.888	0.7891	34.84	26.73	4.738
DT	0.7378	38.38	31.48	5.680	0.7539	37.54	30.68	5.601
RF	0.8276	31.32	25.38	4.562	0.8409	30.22	24.20	4.320
ADBoost	0.8003	33.82	27.66	4.968	0.8358	30.75	24.45	4.374
XGBoost	0.7954	34.28	27.59	4.900	0.7984	33.85	27.04	4.794
SVR	0.8429	29.68	24.20	4.323	0.8810	25.38	20.48	3.668

**Table 6:** Performance after integrating different backbone networks.

Backbone	Pre (%)	IoU (%)	Rec (%)	DSC (%)	Params (M)	Latency (ms)
ResNet50	96.65	94.77	97.98	97.34	26.65	13.77
VGG16	95.88	93.67	97.60	96.82	8.46	8.66
Xception	96.83	94.64	97.67	97.29	23.66	5.92
Ours	97.89	96.35	98.40	98.15	1.51	4.75

**Table 7:** Performance of different combinations of improvement strategies.

MobileNetV3	PMobileNetV3	ARM	CAM	FFM	IoU (%)	DSC (%)
√	-	-	-	-	95.68	97.80
-	√	-	-	-	95.83	97.89
-	√	√	-	-	96.06	98.00
-	√	√	√	-	96.17	98.07
-	√	√	√	√	96.35	98.15

**Table 8:** Performance of different segmentation models on the new dataset.

Model	Pre (%)	IoU (%)	Rec (%)	DSC (%)
UNet	94.69	91.42	96.36	95.40
TransUNet	94.26	91.01	96.35	95.15
DeepLabV3Plus	93.48	90.08	96.12	94.47
BiSeNet	92.87	90.04	96.73	94.61
LR-ASPP	92.56	89.04	95.89	94.01
Ours	96.60	91.62	94.67	95.53

**Table 9:** The detailed time consumption of the method.

Step	Time
Scan X-ray image	approximately 20 s
Get live weight	approximately 10 s
Preprocess image	9.34 ms
Segment image	4.75 ms
Predict breast muscle weight	1.30 ms
All procedures	approximately 30 s

## Data Availability

The code can be requested from the corresponding authors. The data are not publicly available due to being part of an ongoing study and privacy.
